# Evaluation of Analytical Performances of Magnetic Force-Assisted Electrochemical Sandwich Immunoassay for the Quantification of Carcinoembryonic Antigen

**DOI:** 10.3389/fbioe.2021.798079

**Published:** 2022-01-03

**Authors:** Boo Young Hwang, Eunsoo Kim, Seung-ha Kim, Hyundoo Hwang

**Affiliations:** ^1^ Department of Anesthesia and Pain Medicine, School of Medicine, Pusan National University, Yangsan, South Korea; ^2^ Department of Anesthesia and Pain Medicine, Biomedical Research Institute, Pusan National University Hospital, Busan, South Korea; ^3^ BBB Inc., Seoul, South Korea

**Keywords:** performance evaluation, immunoassay, CEA, MESIA, electrochemistry

## Abstract

Carcinoembryonic antigen (CEA) is a biomarker indicated in different cancers, targeted for quantitative analysis *via* immunoassay. Here we introduce a new technique called magnetic force-assisted electrochemical sandwich immunoassay (MESIA) for determination of CEA level in a drop of human serum using a fully automated point-of-care testing (POCT) device. The analytical performances of the assay are assessed based on precision, accuracy, limit of blank (LoB), limit of detection (LoD) and limit of quantitation (LoQ), linearity, Hook effect, interference, cross-reactivity, and method comparison following the guidelines of the Clinical Laboratory Standards Institute (CLSI). The LoD is 0.50 ng/ml. A linear relationship is shown in the range of 0.5–200 ng/ml. A high dose effect is not seen up to approximately 500,000 ng/ml. The recovery range is from 94.7 to 108.9%. The %CV of run-to-run and within-lab variations are less than 2.04 and 4.41% across the CEA concentrations, respectively, whereas reproducibility is 4.45–6.24%. Method comparison shows that the assay correlates well with the reference device (*R* ^*2*^ = 0.9884). The assay demonstrates acceptable precision, accuracy, LoB, LoD and LoQ, hook effect, linearity, interference, cross-reactivity, and high correlation with its reference device. Thus, the system is suitable for the quantification of CEA in clinical practices with a POCT manner.

## 1 Introduction

CEA is a polysaccharide-protein complex produced by the embryonic intestinal mucous membrane prior to birth. As the serum concentration of CEA can increase in the presence of several types of cancer, such as colorectal (Vernava et al., 1994), gastric (Takahashi et al., 2003), lung (Grunnet and Sorensen 2012), or breast (Park et al., 2008) cancers, CEA has been recognized as a broad-spectrum biomarker for cancer diagnosis and prognosis.

A variety of methods have been conducted for the quantitative detection of CEA, such as enzyme-linked immunosorbent assays (ELISA) (Tobi et al., 1992), fluorescence immunoassays (Wu et al., 2015; Yang et al., 2015), chemiluminescence immunoassays (Lin et al., 2004; Jiang et al., 2013), electrochemiluminescence immunoassays (Wang et al., 2015) and amperometric immunoassays (Li et al., 2011). A common drawback of those methods is that they require multiple steps for washing with large amounts of buffer solution as well as sample preparation steps for isolating plasma or serum. These drawbacks can increase the complexity of assays and are the main reasons why the conventional assays could be conducted only by professionals at clinical laboratories. They also require complicated mechanical and optical systems for automation, which obstruct commercialization of miniaturized and portable quantitative immunoassay platforms.

**FIGURE 1:**
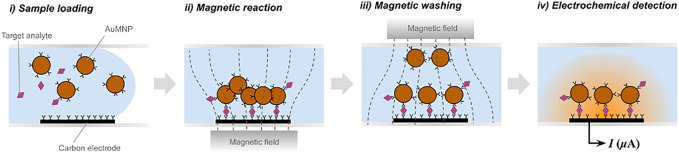
Schematic diagram of magnetic force-assisted electrochemical sandwich immunoassays (MESIA).

Recently, a new immunoassay technique called magnetic force-assisted electrochemical sandwich immunoassay (MESIA) was demonstrated to be suitable for the quantitative detection of serological biomarkers (Hwang et al., 2019b). In MESIA, magnetic nanoparticles capture target analytes and form sandwich immuno-complexes on an electrode surface *via* antibody-antigen interactions (See Figure 1). The mixing and reaction processes are actively controlled by external magnetic fields. Unbound magnetic nanoparticles are removed from the electrode surface by a magnetic field, and the electrochemical signal from the nanoparticles is subsequently measured to determine the concentration of target analytes. This method requires neither a washing buffer nor optical components. Hence, it enables integration of all the assay processes into a single disposable chip and a portable electrochemical reader.

The MARK-B^Ⓡ^ (BBB Inc., Seoul, South Korea) is a point-of-care testing (POCT) immunoassay platform based on the MESIA (Hwang et al., 2019a). The MARK-B immunoassay system is composed of an analyzer, which is an electrochemical reader with a touch-screen mobile device, and a disposable test cartridge that contains gold-coated magnetic nanoparticles (AuMNPs), and screen-printed carbon electrodes (SPCE) in a microfluidic channel. Once a sample is loaded into the cartridge, the capillary force drives the sample into the channel. Subsequently, the pre-spotted AuMNPs are dissolved, and external magnetic fields are actively controlled by two external magnets to facilitate the reaction, in which the antibody-immobilized AuMNPs react with the target analytes to form sandwich immuno-complexes on the SPCE electrochemical sensor. After the reaction process is finalized, unbound AuMNPs are removed by the magnetic force. The amount of target analytes is quantitatively measured by analyzing signals induced by electrochemical oxidation and reduction of gold on the bound AuMNPs—more detailed information regarding the electrochemical measurement technique are described in the previous literature (Hwang et al., 2019b). This technology provides portable, highly sensitive, and fully automated system for quantification of proteins in a drop of liquid specimen without the requirement of any user intervention or additional reagents, thus is relevant for *in vitro* diagnostics based on POCT.

In this study, analytical performances of the MARK-B immunoassay system for the quantitative analysis of CEA are evaluated following the guidelines of the Clinical Laboratory Standards Institute (CLSI). The functional sensitivity, linearity, Hook effect, recovery, precision, and reproducibility of the assay were investigated. In addition, the accuracy was evaluated in comparison with a commercial immunoassay system for hospital uses.

## 2 Materials and Methods

### 2.1 Materials and Reagents

Assay calibrators are traceable to Carcinoembryonic Antigen 1st International Reference Preparation provided by the National Institute of Biological Standards and Controls (NIBSC code: 73/601). Hemoglobin, bilirubin, albumin, gamma globulins, diethylstilbestrol, acetaminophen, acetylsalicylic acid, ampicillin, cyclosporine, goserelin, leuprolide, ibuprofen, ifosfamide, finasteride, flutamide, docetaxel, methotrexate, methyldopa, naproxen, urea, bleomycin, oxaliplatin, triglyceride, vinblastine, and warfarin were purchased from Sigma-Aldrich (Darmstadt, Germany). The beta subunit of human chorionic gonadotropin (Beta-hCG), Alpha-fetoprotein (AFP) and prostate-specific antigen (PSA) from NIBSC; Cancer antigen 19-9 (CA 19-9), cancer antigen 125 (CA-125), human anti-mouse antibody (HAMA) and rheumatoid arthritis (RF) plasma were purchased from Lee Biosolutions (Maryland Heights, MO, United States). Atorvastatin and paclitaxel were purchased from Chemscene (Monmouth Junction, NJ, United States). 5-Fluoro-1- (tetrahydro-2-furfuryl)uracil (Tegafur), doxorubicin, doxycycline, etoposide, furosemide, levodopa, lovastatin, N-acetyl-L-cysteine, prednisone, sodium 2-mercaptoethanesulfonate (Mesna), tamsulosin, theophylline, uric acid, cefoxitin, cisplatin, cyclophosphamide, phenylbutazone, and rifampicin were purchased from TCI (Tokyo, Japan). 5-fluorouracil and L-ascorbic acid were purchased from Biosesang (Seongnam, South Korea).

### 2.2 Clinical Sample Preparation

From the November 28, 2019 to the December 13, 2019, 140 serum samples from colorectal cancer patients were collected from biorepositories. The serum samples were separated by centrifugation and stored at -70°C with complete storage records and were freeze-thawed prior to being used. All the information of the samples were anonymous until the end of detection.

### 2.3 Instruments

MARK-B (BBB Inc., Seoul, South Korea) and Unicel DxI 800 ACCESS Immunoassay System (Beckman Coulter, Indianapolis, IN, United States) as the reference device were applied for this study.

### 2.4 Functional Sensitivity: Limit of Blank, Limit of Detection, and Limit of Quantitation

The limit of blank (LoB) and the limit of detection (LoD) were determined according to the CLSI guideline EP-17-A2 (Clinical and Laboratory Standards Institute (CLSI) guidelines, 2012). The mean and standard deviation were calculated *via* 30 consecutive measurements of blank and low concentration specimens each. To assess the limit of quantitation (LoQ), the coefficients of variation (CVs) for specimens with various CEA concentrations were calculated. Each measurement was performed in 20 replicates. The lowest concentration measured with a CV ≤ 15% was defined as the estimated LoQ.

### 2.5 Linearity

Linearity was evaluated according to CLSI guideline EP-6-A (Clinical and Laboratory Standards Institute (CLSI)guidelines 2003). A sample with high analyte concentration (200 ng/ml) was diluted with one with low analyte concentration (0.5 ng/ml) into nine different fractional parts of each sample—0.50, 25.44, 50.38, 75.31, 100.25, 125.19, 150.13, 175.06, and 200.00 ng/ml. Five replicates were measured for each dilution fold using a single lot of cartridges. Linear regression analysis was conducted as recommended by Kroll and Emancipator (Kroll and Emancipator 1993).

### 2.6 Hook Effect

A specimen with CEA concentration of 500,000 ng/ml and its serial dilution samples each was used to assess the hook effect with the CEA assay. The specimen was diluted with a serum containing CEA below the assay detection limit, and each measurement was performed in four replicates.

### 2.7 Recovery

Four different CEA concentration samples - high (151.4 ng/ml), higher mid (75.2 ng/ml), and lower mid (15.5 ng/ml) and low (3.42 ng/ml)—were measured in four replicates. The percentage ratio between the measured and estimated concentrations was calculated. The acceptance criteria set for this study was a recovery range between 90–110%.

### 2.8 Single-Site Precision

Precision was evaluated according to CLSI guideline EP5-A (Clinical and Laboratory Standards Institute (CLSI) guidelines 2004). Human serum pools with five different levels of CEA concentration for each assay were obtained—Level 1 (0.5–2.00 ng/ml), Level 2 (2.00–10.0 ng/ml), Level 3 (10.0–50.0 ng/ml), Level 4 (50.0–100 ng/ml), and Level 5 (100.0–200.0 ng/ml). To determine and estimate repeatability, between-run, between-day and within-lab imprecisions, the five levels were measured in two replicates per run, two runs per day over 20 days using a single lot. Between-day imprecision was evaluated based on 40 runs over 20 separate days. The acceptance criteria were predetermined to be that repeatability, between-run, between-day and within-lab imprecisions are all less than 15%.

### 2.9 Multi-Site Precision

Human serum pools with five different levels of CEA concentration for each assay were obtained—Level 1 (0.5–2.00 ng/ml), Level 2 (2.00–10.0 ng/ml), Level 3 (10.0–50.0 ng/ml), Level 4 (50.0–100 ng/ml), and Level 5 (100.0–200.0 ng/ml). The five levels were measured in five replicates per day; 5 days per lot; two lots per site at three different sites. In the end, the data is statistically analyzed to determine and estimate the repeatability, between-day, between-lot, between-site and reproducibility imprecisions. The acceptance criteria were predetermined to be that repeatability, between-day and total variations are all less than 10%.

### 2.10 Interference Tests

Interference was evaluated according to CLSI guideline EP07-A2 (Clinical and Laboratory Standards Institute (CLSI) guidelines 2008), by testing drug free specimens at two CEA concentrations prepared in CEA-negative human sera (0.5–10.0 ng/ml and 100.0–200.0 ng/ml), spiked with potential interferents: hemoglobin (500 mg/dl), hemoglobin (1,000 mg/dl); bilirubin (20 μg/ml); bilirubin (60 mg/ml); total protein (5 g/dl); total protein (12 g/dl); triglyceride (3 g/dl); HAMA (52.5 ng/ml); RF (500 IU/ml), flutamide (10 μg/ml), diethylstilbestrol (5 μg/ml), goserelin (40 ng/ml); acetaminophen (250 ng/ml); acetysalicylic acid (600 μg/ml); leuprolide (275 ng/ml); ibuprofen (500 μg/ml); finasteride (250 ng/ml); docetaxel (10 μg/ml); urea (500 mg/dl); uric acid (20 mg/dl); 5-fluorouracil; ampicilin (1 mg/ml); ascorbic acid (300 μg/ml); astorvastatin (3,000 μg/ml); bleomycin (3 mg/dl); cefoxitin (2.5 mg/ml); cisplatin (8.8 mg/dl); cyclophosphamide (327.9 mg/dl); cyclosporine (10 μg/ml); diethylstilbestrol (5 μg/ml); docetaxel (10 μg/ml); doxorubicin (16.5 mg/dl); doxycycline (50 μg/ml); etoposide (22 mg/dl); finasteride (250 ng/ml); flutamide (10 μg/ml); furosemide (4 mg/ml); goserelin (40 ng/ml); ibuprofen (500 μg/ml); ifosfamide (261.8 mg/dl); leuprolide acetate (275 ng/ml); levodopa (20 μg/ml); lovastatin (2.5 μg/ml); Mesna (84 mg/dl); methotrexate (459.5 mg/dl); methyldopa (20 μg/ml); N-acetyl-L-cysteine (150 μg/ml); naproxen (500 μg/ml); oxaliplatin (100 μg/ml); paclitaxel (38.2 mg/dl); phenylbutazone (400 μg/ml); prednisone (5 μg/ml); rifampicin (60 μg/ml); tamsulosin (100 ng/ml); tegafur (50 μg/ml); theophylline (50 μg/ml); vinblastine (4 mg/dl); vincristine (0.44 mg/dl); and warfarin (50 μg/ml). The potential interferents and CEA specimens were mixed at a ratio of one part to 19 parts, respectively (1-in-20 dilution) to prepare the test samples. Control sample was also prepared by diluting another aliquot of the same CEA specimen with pure solvent or a diluting solution without any suspected interferents. The percentage interference was calculated from the difference in mean CEA concentration between the test sample and the control sample. If the absolute value of percentage interference was less than 15%, then the assay is deemed to have 100 ± 15% recovery with no interference to the substances.

### 2.11 Cross-Reactivity Tests

Specimens spiked with beta-hCG (206 mIU/ml), CA125 (100 IU/ml), CA19-9 (423 U/mL), AFP (500 ng/ml), and PSA (50 ng/ml) were prepared to evaluate cross-reactivity. Specimens at two CEA concentrations (identical to the those indicated in Section 2.10) were tested for each potential cross-reacting compound in three replicates, and the percentage bias was calculated.

### 2.12 Method Comparison

The method comparison was conducted according to CLSI guideline EP09-A2 (Clinical and Laboratory Standards Institute (CLSI) guidelines 2013) at the EONE Laboratories (Incheon, South Korea). 140 human serum samples evenly distributed across the entire measuring range were collected from biorepositories, and stored at −70°C before analysis. The samples were analyzed using the Unicel DxI 800 Access Immunoassay System (Beckman Coulter) as the reference device. All specimens were also analyzed on a MARK-B immunoassay system for comparison. Deming regression analysis was conducted to define the Pearson correlation coefficient and the 95% confidence interval (CI) for both the proportional bias (slope) and constant bias (intercept) were determined to claim whether each is significantly different from 1.0 to 0, respectively.

## 3 Results

### 3.1 Functional Sensitivity: Limit of Blank, Limit of Detection, and Limit of Quantitation

The claimed LoB was 0.47 ng/ml, where 28 out of 30 blank measurements (93%) were less than or equal to the LoB claim, which is higher than the lower bound (87%) for the sample size of 30. Therefore, the LoB claim was successfully verified. The claimed LoD was 0.50 ng/ml, where 27 out of 30 low level sample measurements (90%) were greater than or equal to the LoB claim. Therefore, the LoD claim was successfully verified. The claimed LoQ was 0.50 ng/ml, where 28 out of 30 measurements (93%) fell within the allowable error window. Therefore, the LoQ claim was successfully verified and was determined to be the same as the LoD.

### 3.2 Linearity

**FIGURE 2:**
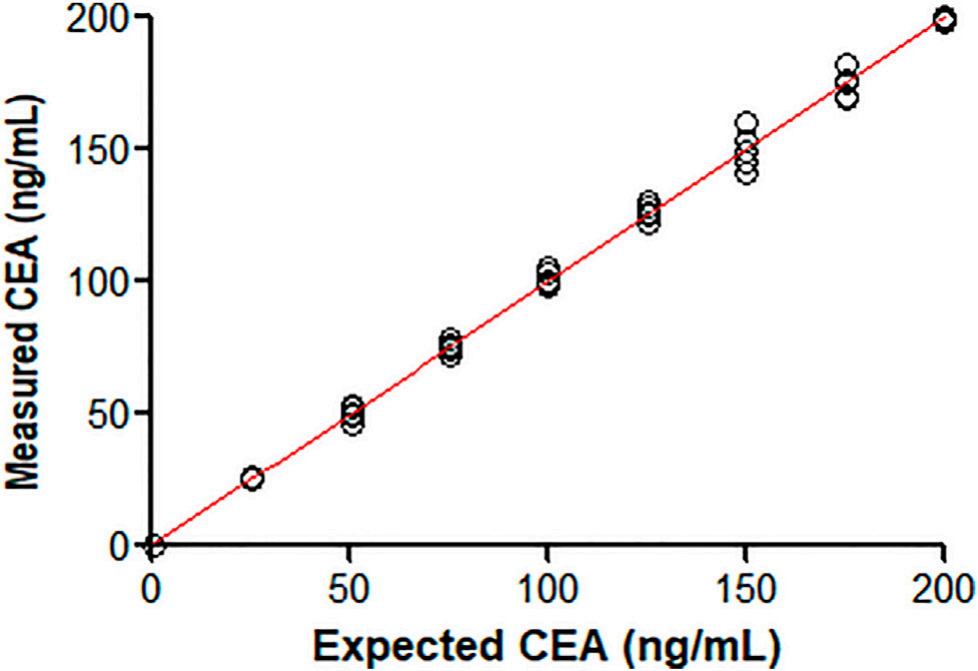
Comparison of measured CEA values against known concentrations of nine different CEA-spiked samples.

The measured CEA values of the diluted samples are shown in Figure 2. The line of best fit drawn in Figure 1 produced the following equations as a first-order, second-order and third-order polynomial regression: the first order, *y* = 0.9971*x*+ 0.3256; the second order, *y* = −9.498e⁻⁵*x* ^2^+1.016*x*−0.2352; the third order, *y* = −6.52e^−⁷^ *x* ^3^+0.0001011*x* ^2^+1.001*x*−0.05795. The nonlinear coefficients of the second-order and the third-order models, *b*_2_ and *b*_3_, were not statistically significant; that is, the coefficients were not significantly different from 0. Therefore, the dataset was considered linear from 0.5 to 200 ng/ml.

### 3.3 Hook Effect

**FIGURE 3:**
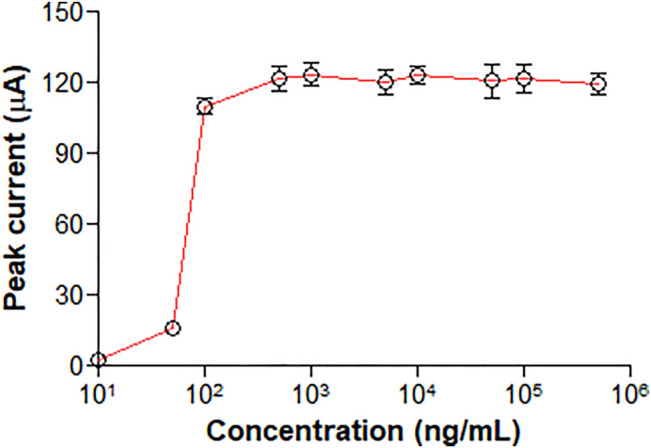
Representation of peak current over a concentration range from 10 to 500,000 ng/ml CEA.

No clear high dose hook effect was observed up to approximately 500,000 ng/ml, as shown in Figure 3.

### 3.4 Recovery

As shown in Table 1, the %recovery ranged from 94.7 to 108.9% with a mean of 100.3%. The recovery range satisfied the predetermined acceptance criteria.

**TABLE 1:** Mean % recoveries evaluated from four replicates for each level of concentration.

Sample	Expected concentration (ng/ml)	Measured concentration (ng/ml)	%Recovery	Mean %Recovery
Level 1	3.42	3.30	96.5	99.9
		3.61	105.6	
		3.24	94.7	
		3.51	102.6	
Level 2	15.50	14.90	96.1	99.7
		16.20	104.5	
		15.60	100.6	
		15.10	97.4	
Level 3	75.20	72.30	96.1	102.0
		78.50	104.4	
		81.90	108.9	
		74.20	98.7	
Level 4	151.40	151.20	99.9	99.6
		146.20	96.6	
		149.90	99.0	
		155.70	102.8	

### 3.5 Single-Site Precision Study

The %CV for repeatability ranged from 3.32 to 4.45% (See Table 2). The %CV for run-to-run and day-to-day variations ranged from 0 to 2.04% and 0–1.71%, respectively, across the CEA concentrations from 1.3 ng/ml to 154.9 ng/ml. The within-lab variability across all samples ranged less than 4.41% across the CEA concentrations. Since all single-site precision %CV values were significantly less than 15%, the assay was deemed to satisfy the acceptance criteria.

**TABLE 2:** Statistical analysis for single-site precision.

Sample	Mean (ng/ml)	N	Repeatability		Between-run		Between-day		Within-lab	
			SD	%CV	SD	%CV	SD	%CV	SD	%CV
1	1.30	80	0.058	4.45	0.000	0.00	0.000	0.00	0.053	4.06
2	3.35	80	0.138	4.11	0.000	0.00	0.038	1.13	0.137	4.10
3	15.3	80	0.539	3.52	0.312	2.04	0.263	1.71	0.676	4.41
4	74.7	80	2.478	3.32	1.228	1.64	0.750	1.00	2.866	3.84
5	154.9	80	5.372	3.47	2.698	1.74	0.604	0.39	4.606	2.97

### 3.6 Multisite Precision Study

The %CV for repeatability ranged from 4.21 to 6.63% (See Table 3). The %CV for between-day, between-lot and between-site variations were less than 3.28% across all the sites, lots, days and CEA concentrations. The %CV for reproducibility ranged from 4.45 to 6.24%. Since all %CV values for repeatability, between-day, between-lot, between-site and reproducibility were less than 10%, the assay was deemed to be acceptable.

**TABLE 3:** Statistical analysis for multi-site precision.

Sample	Mean (ng/ml)	N	Repeatability		Between-day		Between-lot		Between-site		Reproducibility	
			SD	%CV	SD	%CV	SD	%CV	SD	%CV	SD	%CV
1	1.29	150	0.096	6.63	0.016	1.22	0.000	0.00	0.004	0.32	0.081	6.24
2	3.34	150	0.141	4.21	0.028	0.84	0.093	2.77	0.000	0.00	0.149	4.45
3	15.50	150	0.872	5.62	0.000	0.00	0.201	1.30	0.000	0.00	0.844	5.44
4	75.90	150	3.473	4.58	0.991	1.31	1.596	2.10	0.906	1.19	3.813	5.02
5	158.20	150	6.971	4.41	5.186	3.28	3.330	2.10	1.441	0.91	7.838	4.95

### 3.7 Interference Tests

The total interference (%) only ranged between −8.01 and 10.28% for both endogenous serum substances (See Supplementary Table S1) and drug substances (See Supplementary Table S2). Therefore, no significant interference was observed from neither the tested drugs nor the endogenous serum substances that would affect the interpretation of CEA results in this assay. No cross-reactivity with beta-hCG, CA 125, CA 19-9, AFP, and PSA was also observed as shown in Supplementary Table S3.

### 3.8 Method Comparison

**FIGURE 4:**
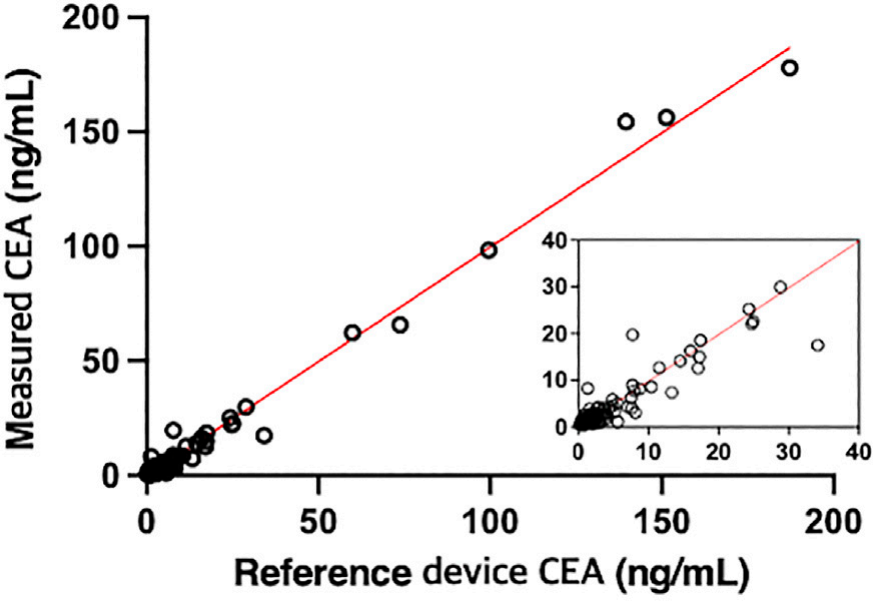
Comparison of the CEA values of 131 samples derived from patients measured by the proposed method with those measured by a reference device, UniCel DxI 800 Access Immunoassay System (Beckman Coulter) (*r* = 0.9942, *y* = 0.9985*x* - 0.1924).

140 samples were tested with the proposed and reference methods, nine of which were out of range (<0.5 or >200 ng/ml). Deming regression analysis of comparison gave a slope of 0.9985 (95% CI 0.9798–1.017) and an intercept of −0.1924 (95% CI −0.7154 to 0.3305) as shown in Figure 4 and Supplementary Table S4. The slope was not significantly different from 1.0 (95% CI of slope includes 1.0) indicating the lack of proportional bias in assay results between the proposed and reference methods. The intercept was not significantly different from 0 (95% CI includes 0) indicating the lack of constant bias between the two methods. *R* (Takahashi et al., 2003) was 0.9884 indicating the differences between the proposed and reference methods are small enough.

## 4 Discussion

Highly elevated concentrations of CEA in the blood have been known to be associated with a variety of different cancers including colon cancer, stomach cancer, large intestinal cancer (90%), non-small-cell lung carcinoma (70%) and breast cancer (50%) (Thompson et al., 1991). CEA is known to be produced on the cancer cell surface and distributed into the bloodstream, weakening immune responses and inducing cancer cell metastasis (Konstadoulakis et al., 1994; Thomas et al., 1995; Haidopoulos et al., 2000). Therefore, monitoring and managing blood CEA concentration are highly important in managing the conditions of cancer patients. Through a variety of studies, the importance of monitoring blood CEA concentration has been consolidated: it could be used as an indicator to predict whether the cancer would grow and spread again after surgery and make other prognostic assessments (Moertel et al., 1993); predict the survival and death rate (Ebeling et al., 2002). It has also been reported to have a sensitivity of 69% and a specificity of 68% in diagnosing lung cancer (Okamura et al., 2013).

Recently, the demand for not only within-laboratory, but also “in-the-field” (i.e., in a natural environment outside of hospitals and laboratories) diagnostic tests for better access to *in vitro* diagnostic instruments has been continuously expanding. As the *in vitro* diagnostic instruments being used in clinical laboratories and hospitals are too large and heavy to transport and complicated in their analyses requiring the assistance of an expert and other instruments to operate, diagnosis in the field is impossible. Hence, to be able to conduct diagnosis in the field, the instrument must not only be accurate and precise, but should also demonstrate portability and automation.

Currently, the most commonly adopted immunoassay techniques in POCT is lateral flow immunoassay (LFIA) (Liu et al., 2016; Lee and Lee 2020; Mahmoudi et al., 2021). In the case of LFIA, accurate quantification of protein concentration is very difficult, since the LFIA is based on a paper sheet, which is a random fiber network, to deliver the samples and reagents to the detection site. The flow through the paper sheet is also dependent on many physical parameters, such as the temperature, the gravity, the viscosity of samples, and so on. The antibody-antigen reactions in the LFIA are passive, thus its performances are not tunable once established. Due to the intrinsic limitations, most of the commercial rapid diagnostic testing kits based on LFIA could be applied only for the qualitative analysis of biomarkers, rather than the quantitative analysis.

The MESIA resolves all the issues mentioned prior. The antigen-antibody reactions are facilitated and actively controlled using AuMNPs and external magnetic fields (Hwang et al., 2019b). Therefore, the time for analysis is relatively short and the reaction scheme is programmable for the optimization of reaction conditions depending on the target biomarkers. The required volume of the sample is only a few microliters, which is fixed by the volume of the reaction chamber that is a fine plastic microchannel confined by a microvalve. The MESIA doesn’t require any washing buffer, but utilizes the magnetic fields to remove unbound proteins and probes, thus all the required functions for immunoassay can be integrated into a tiny disposable chip. The detection is based on electrochemistry, so no optical components are required for a reader, which makes it portable and cost-effective, resulting in an *in vitro* diagnostic device relevant for POCT.

In this study, performance evaluation of the MARK-B immunoassay system, which is based on the MESIA, has been conducted according to the CLSI guidelines, where analytical parameters including linearity, hook effect, precision, recovery, interference, cross-reactivity, and accuracy compared to the reference device were assessed. The MARK-B immunoassay system satisfied all the acceptance criteria for the analytical performances. Moreover, the method comparison results with Unicel DxI 800 Access Immunoassay System (Beckman Coulter) as the reference device revealed an *R* (Takahashi et al., 2003) value of 0.9985, showing that the CEA measurand values of the MESIA-incorporated instrument are almost equivalent to those of the large-scale instrument.

In the MARK-B immunoassay system, all the processes from sample preparation to electrochemical detection are fully integrated and automated, as like conventional automated instruments for hospital uses. Most of the automated instrument for CEA immunoassay utilize chemiluminescence immunoassay (Matsushita et al., 1996; Akbas et al., 2014; Zhang et al., 2016; Park et al., 2018), resulting in high sensitivity and high throughput, but they are too heavy and large to be mobile for the POCT applications. Although lots of research on the lab-on-a-chip technology that targets CEA for POCT have been reported in the past (e.g., glass capillaries (Hu et al., 2013), a combination of microfluidics and nanoimprint with plasmonic biochips (Zhou et al., 2019), and electrochemical microfluidic chip (Xie et al., 2015)), most of the currently being researched lab-on-a-chip techniques do not address the issues regarding mass production and reproducibility. The MARK-B immunoassay system has already been successfully validated and commercialized for both qualitative and quantitative determination of various biomarkers by applying different types of antibodies, proving that the platform could be useful for the diagnostics of a variety of diseases including infectious diseases and cancers (Hwang et al., 2019a; Jo et al., 2021).

In conclusion, the MARK-B immunoassay system, which is based on MESIA, satisfied the acceptable criteria for precision, accuracy, functional sensitivity, Hook effect, linearity, interference, cross-reactivity, and method comparison with a commercial instrument for hospital uses. Therefore, the MARK-B immunoassay system has been shown to be useful in clinical practices as a rapid, accurate, and convenient way for the quantification of CEA.

## Data Availability

The original contributions presented in the study are included in the article/Supplementary Material, further inquiries can be directed to the corresponding author.
