# Supplementary material for: Evaluation of Analytical Performances of Magnetic Force-Assisted Electrochemical Sandwich Immunoassay for the Quantification of Carcinoembryonic Antigen
Source: Front Bioeng Biotechnol. 2022 Jan 3;9:798079. doi: 10.3389/fbioe.2021.798079 (PMC8767762; doi:10.3389/fbioe.2021.798079)
Supplement: Supplementary file 1 [file DataSheet1.docx]

**Supplementary Information**

**Evaluation of analytical performances of magnetic force-assisted electrochemical sandwich immunoassay for the quantification of carcinoembryonic antigen**

Boo Young Hwang^1^, Eunsoo Kim^1^, Seung-ha Kim^1^, Hyundoo Hwang^2*^

^1^Department of Anesthesia and Pain Medicine, Pusan National University Hospital, Busan, Republic of Korea

^2^BBB Inc., Seoul, Republic of Korea

**Correspondence**

Hyundoo Hwang, BBB Inc., Seoul, Republic of Korea

Email: doo@bbbtech.com

**1. Interference tests**

The total interference (%) only ranged between -8.01% and 10.28% for both endogenous serum substances (See Table S1) and drug substances (See Table S2). Therefore, no significant interference was observed from neither the tested drugs nor the endogenous serum substances that would affect the interpretation of CEA results in this assay. No cross-reactivity with beta-hCG, CA 125, CA 19-9, AFP, and PSA was also observed as shown in Table S3.

Table S1. Interference from endogenous serum substances

| Substances | Concentration tested | Control (Low) | Test (Low) | %Interference | Control (High) | Test (High) | %Interference |
| --- | --- | --- | --- | --- | --- | --- | --- |
| Albumin | 150 g/L | 3.50 | 3.46 | -1.14 | 151.0 | 158.0 | 4.64 |
| Hemoglobin | 500 mg/dL | 3.78 | 3.64 | -3.70 | 166.9 | 167.7 | 0.48 |
| Hemoglobin | 1,000 mg/dL | 3.98 | 3.75 | -5.78 | 162.9 | 154.8 | -4.97 |
| Bilirubin | 20 mg/dL | 3.78 | 3.73 | -1.32 | 163.3 | 168.5 | 3.18 |
| Bilirubin | 60 mg/dL | 3.89 | 3.77 | -3.08 | 161.0 | 164.6 | 2.24 |
| Total protein | 5 g/dL | 3.74 | 3.78 | 1.07 | 166.3 | 155.7 | -6.37 |
| Total protein | 12 g/dL | 3.62 | 3.66 | 1.10 | 175.3 | 162.2 | -7.47 |
| Triglyceride | 3 g/dL | 3.81 | 3.81 | 0.00 | 170.4 | 163.3 | -4.17 |
| HAMA | 52.5 ng/mL | 3.70 | 3.64 | -1.62 | 161.2 | 150.2 | -6.82 |
| RF | 500 IU/mL | 3.82 | 3.69 | -3.40 | 165.7 | 166.3 | 0.36 |
| Urea | 500 mg/dL | 3.64 | 3.76 | 3.30 | 160.9 | 167.7 | 4.23 |
| Uric Acid | 20 mg/dL | 3.92 | 3.77 | -3.83 | 175.2 | 164.8 | -5.94 |

Table S2. Interference from drug substances

| Substances | Concentration tested | Control (Low) | Test (Low) | %Interference | Control (High) | Test (High) | %Interference |
| --- | --- | --- | --- | --- | --- | --- | --- |
| 5-fluorouracil | 280 μg/mL | 3.83 | 3.77 | -1.57 | 170.3 | 175.2 | 2.88 |
| Acetaminophen | 250 ng/mL | 3.96 | 3.68 | -7.07 | 169.4 | 166.1 | -1.95 |
| Acetylsalicylic acid | 600 μg/mL | 3.82 | 3.79 | -0.79 | 159.8 | 153.8 | -3.75 |
| Ampicillin | 1 mg/mL | 3.73 | 3.81 | 2.14 | 163.1 | 170.1 | 4.29 |
| Ascorbic acid | 300 μg/mL | 3.87 | 3.56 | -8.01 | 156.7 | 171 | 9.13 |
| Atorvastatin | 3 μg/mL | 3.67 | 3.93 | 7.08 | 170.3 | 173.5 | 1.88 |
| Bleomycin | 3.3 mg/dL | 3.79 | 3.81 | 0.53 | 169.1 | 165.4 | -2.19 |
| Cefoxitin | 2.5 mg/mL | 3.91 | 3.80 | -2.81 | 155.6 | 169.3 | 8.80 |
| Cisplatin | 8.8 mg/dL | 3.90 | 3.84 | -1.54 | 170.7 | 173.2 | 1.46 |
| Cyclophosphamide | 327.9 mg/dL | 3.83 | 3.89 | 1.57 | 170.8 | 168.5 | -1.35 |
| Cyclosporine | 10 μg/mL | 3.90 | 3.87 | -0.77 | 172.4 | 163.9 | -4.93 |
| Diethylstilbestrol | 5 μg/mL | 3.72 | 3.78 | 1.61 | 164.5 | 161.7 | -1.70 |
| Docetaxel | 10 μg/mL | 3.86 | 3.70 | -4.15 | 163.8 | 164.9 | 0.67 |
| Doxorubicin | 16.5 mg/dL | 3.49 | 3.84 | 10.03 | 167.2 | 162.7 | -2.69 |
| Doxycycline | 50 μg/mL | 3.75 | 3.86 | 2.93 | 174.5 | 168.6 | -3.38 |
| Etoposide | 22 mg/dL | 3.80 | 3.80 | 0.00 | 174.2 | 167.1 | -4.08 |
| Finasteride | 250 ng/mL | 4.03 | 3.71 | -7.94 | 168.2 | 166.5 | -1.01 |
| Flutamide | 10 μg/mL | 3.90 | 3.79 | -2.82 | 173.4 | 168.3 | -2.94 |
| Furosemide | 4 mg/mL | 3.73 | 3.78 | 1.34 | 167.2 | 166.9 | -0.18 |
| Goserelin | 40 ng/mL | 3.99 | 3.80 | -4.76 | 168.2 | 169.5 | 0.77 |
| Ibuprofen | 500 μg/mL | 3.79 | 3.82 | 0.79 | 165.8 | 171.9 | 3.68 |
| Ifosfamide | 261.8 mg/dL | 4.03 | 3.76 | -6.70 | 162.5 | 171.7 | 5.66 |
| Leuprolide | 275 ng/mL | 3.86 | 3.69 | -4.40 | 160.8 | 171.5 | 6.65 |
| Levodopa | 20 μg/mL | 3.78 | 3.87 | 2.38 | 166.1 | 176.4 | 6.20 |
| Lovastatin | 2.5 μg/mL | 3.76 | 3.76 | 0.00 | 167.8 | 166.3 | -0.89 |
| Mesna | 84 mg/dL | 3.80 | 3.75 | -1.32 | 161.6 | 164.2 | 1.61 |
| Methotrexate | 459.5 mg/dL | 3.88 | 3.83 | -1.29 | 162.4 | 179.1 | 10.28 |
| Methyldopa | 20 μg/mL | 3.74 | 3.89 | 4.01 | 174.4 | 167.9 | -3.73 |
| N-acetyl-L-cysteine | 150 μg/mL | 3.76 | 3.90 | 3.72 | 178.1 | 170.2 | -4.44 |
| Naproxen | 500 μg/mL | 3.71 | 3.73 | 0.54 | 163 | 163.9 | 0.55 |
| Oxaliplatin | 100 μg/mL | 3.77 | 3.77 | 0.00 | 166.7 | 168.4 | 1.02 |
| Paclitaxel | 38.2 mg/dL | 3.76 | 3.92 | 4.26 | 173.7 | 168.8 | -2.82 |
| Phenylbutazone | 400 μg/mL | 3.77 | 3.69 | -2.12 | 177.2 | 167.2 | -5.64 |
| Prednisone | 5 μg/mL | 3.87 | 3.67 | -5.17 | 168.6 | 170.5 | 1.13 |
| Rifampicin | 60 μg/mL | 3.86 | 4.00 | 3.63 | 171.3 | 168.8 | -1.46 |
| Tamsulosin | 100 ng/mL | 3.75 | 3.87 | 3.20 | 168.8 | 170.1 | 0.77 |
| Tegafur | 50 μg/mL | 3.83 | 3.90 | 1.83 | 166.7 | 172.7 | 3.60 |
| Theophylline | 50 μg/mL | 3.73 | 3.69 | -1.07 | 163 | 163.6 | 0.37 |
| Vinblastine | 4 mg/dL | 3.65 | 3.99 | 9.32 | 163.8 | 164.4 | 0.37 |
| Vincristine | 0.44 mg/dL | 3.90 | 3.83 | -1.79 | 158.7 | 159.5 | 0.50 |
| Warfarin | 50 μg/mL | 3.92 | 3.78 | -3.57 | 162.5 | 170.9 | 5.17 |

Table S3. Cross-reactivity between antigens

| Antigen tested | Concentration tested | Control (Low) | Test (Low) | %Interference | Control (High) | Test (High) | %Interference |
| --- | --- | --- | --- | --- | --- | --- | --- |
| Beta-hCG | 206 mIU/mL | 3.69 | 3.74 | 1.36 | 161.1 | 167.7 | 4.10 |
| CA125 | 100 IU/mL | 3.81 | 3.69 | -3.15 | 170.9 | 162.6 | -4.86 |
| CA19-9 | 423 U/mL | 3.91 | 3.67 | -6.14 | 166.8 | 176.1 | 5.58 |
| AFP | 500 ng/mL | 3.77 | 3.90 | 3.45 | 166.6 | 157 | -5.76 |
| PSA | 50 ng/mL | 3.88 | 3.78 | -2.58 | 166.3 | 169.2 | 1.74 |

**2. Method comparison**

140 samples were tested with the proposed and reference methods, 9 of which were out of range (<0.5 or >200 ng/mL). Deming regression analysis of comparison gave a slope of 0.9985 (95% CI 0.9798 to 1.017) and an intercept of -0.1924 (95% CI -0.7154 to 0.3305) as shown in Table S4. The slope was not significantly different from 1.0 (95% CI of slope includes 1.0) indicating the lack of proportional bias in assay results between the proposed and reference methods. The intercept was not significantly different from 0 (95% CI includes 0) indicating the lack of constant bias between the two methods. *R*² was 0.9884 indicating the differences between the proposed and reference methods are small enough.

Table S4. Statistical analysis of regression

| Slope  (95 % CI) | *y*-intercept when *y*=0 (95% CI) | *x*-intercept when *y*=0 (95% CI) | 1/slope | *R*² |
| --- | --- | --- | --- | --- |
| 0.9985 ± 0.0095 (0.9798‒1.017) | -0.1924 ± 0.2668  (-0.7154‒0.3305) | 0.1927 (-0.3331‒0.7122) | 1.002 | 0.9884 |
